# Unusual metastasis of papillary renal cell carcinoma to the pyriform sinus: Case report

**DOI:** 10.1002/ccr3.2162

**Published:** 2019-05-14

**Authors:** Edoardo Gioia, Filippo Carta, Cinzia Mariani, Clara Gerosa, Roberto Puxeddu

**Affiliations:** ^1^ Unit of Otorhinolaryngology, Department of Surgery Azienda Ospedaliero‐Universitaria di Cagliari, University of Cagliari Cagliari Italy; ^2^ Unit of Pathology, Department of Surgery Azienda Ospedaliero‐Universitaria di Cagliari, University of Cagliari Cagliari Italy

**Keywords:** CO_2_ laser, endoscopy, larynx, metastasis, renal cell carcinoma, transoral microsurgery

## Abstract

Renal cell carcinoma is the third most common cause of distant metastasis to the head and neck. Renal cell carcinoma metastasis should be considered in differential diagnosis when patients with a clinical history of renal cell carcinoma show a head and neck mass.

## 1 INTRODUCTION

Renal cell carcinoma (RCC), also known as hypernephroma, is the malignancy with the most unusual and unpredictable behavior.^1^ RCC can metastasize to the head and neck region, showing a clinical presentation similar to a primary lesion^1^ but, although 8%‐15% of RCC extracranial metastasis are observed in head and neck, hypopharyngeal localizations are uncommon. We report the clinical case of a papillary RCC (pRCC) presenting as hypopharyngeal pedicled mass in an elderly male patient who was referred to our Unit from the Emergency department for hemoptysis, dyspnea, and dysphagia.

## 2 CASE HISTORY

**Figure 1:**
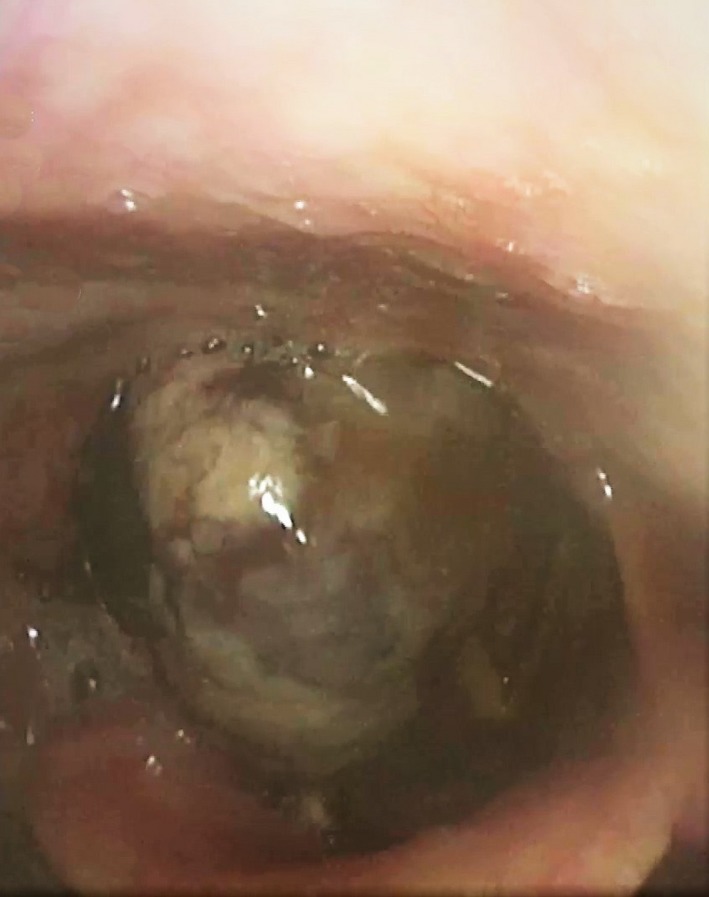
Endoscopic view of a large pedicled lesion originating from the right pyriform sinus with severe impairment of the airway

**Figure 2:**
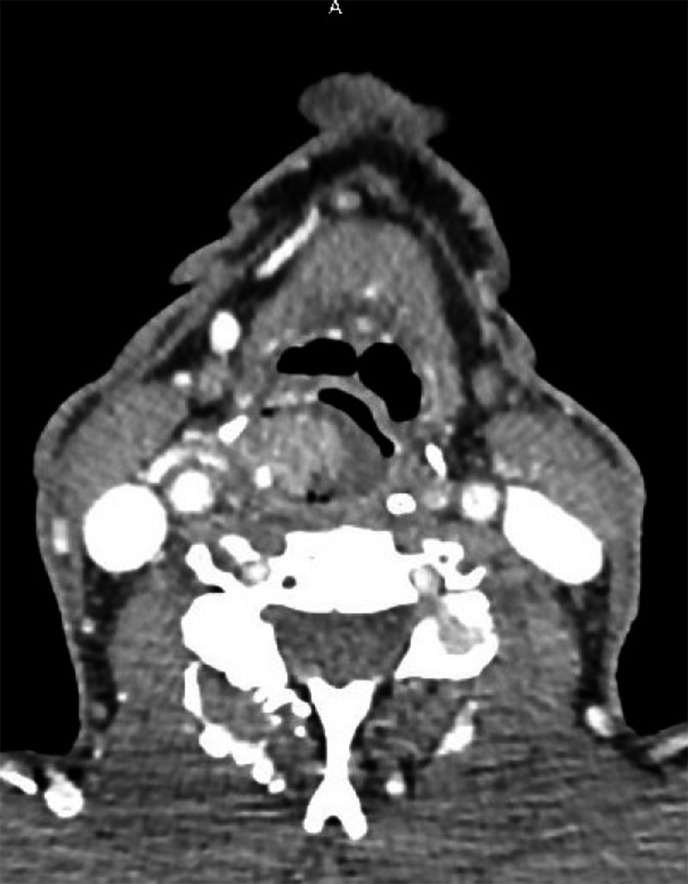
CT scan of the neck with contrast medium showing an oval mass of 25 mm of the right pyriform sinus

An 84‐year‐old man was admitted to the Unit of Otorhinolaryngology of the University Hospital of Cagliari in October 2018 for a 10‐day symptomatology characterized by dysphagia, odynophagia, globus pharyngeus, and occasional hemoptysis. The patient had a clinical history of previous treatment with pazopanib for a cT3N1M0, stage group III pRCC diagnosed in March 2018, and he also was a stroke survivor. Fibrolaryngoscopy showed a large pedicled lesion originating from the right pyriform sinus extended to the laryngeal vestibule with severe impairment of the respiratory space (Figure 1). CT scan of neck, chest, and abdomen with contrast medium showed the presence of an oval mass of 25 mm of major axis of the right pyriform sinus and the presence of the pRCC of the left kidney (64 × 82 mm) associated with conglomerate para‐aortic nodes (63 mm; Figure 2).

The patient underwent radical resection through transoral CO_2_ laser microsurgery (TLM). The procedure was performed under general anesthesia through orotracheal intubation with a Mallinckrodt tube Athlone, Ireland. The following microscope was used during the surgical procedure: Zeiss Universal S2 (Jena, Germany) with 400 mm focal lens coupled with an Acupulse (Tel Aviv, Israel) CO_2_ laser with an Acublade (Tel Aviv, Israel) focusing system. The super‐pulse mode was used at 10 watts, shape size 1‐2 mm, depth 2, continuous wave.

**Figure 3:**
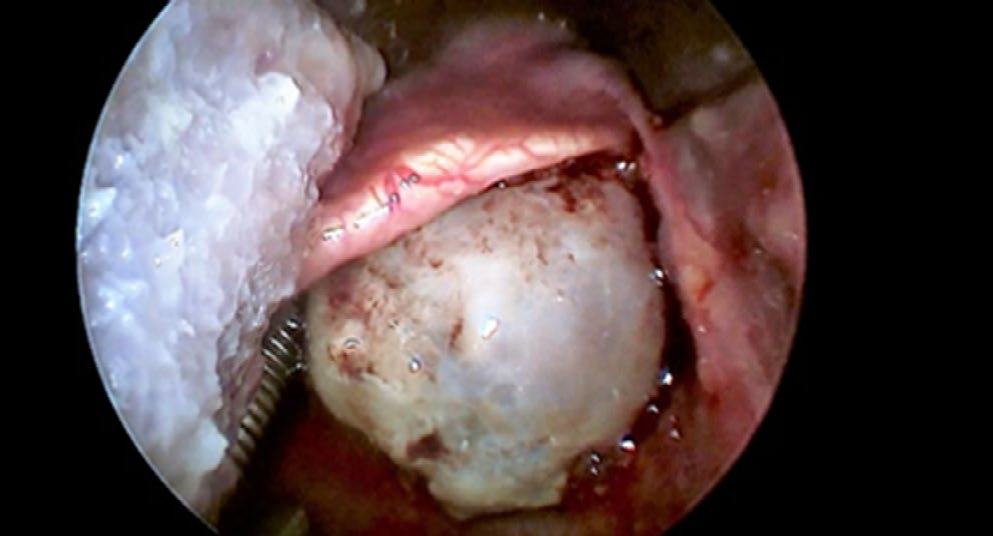
Intraoperative view of the lesion originating from the right pyriform sinus

**Figure 4:**
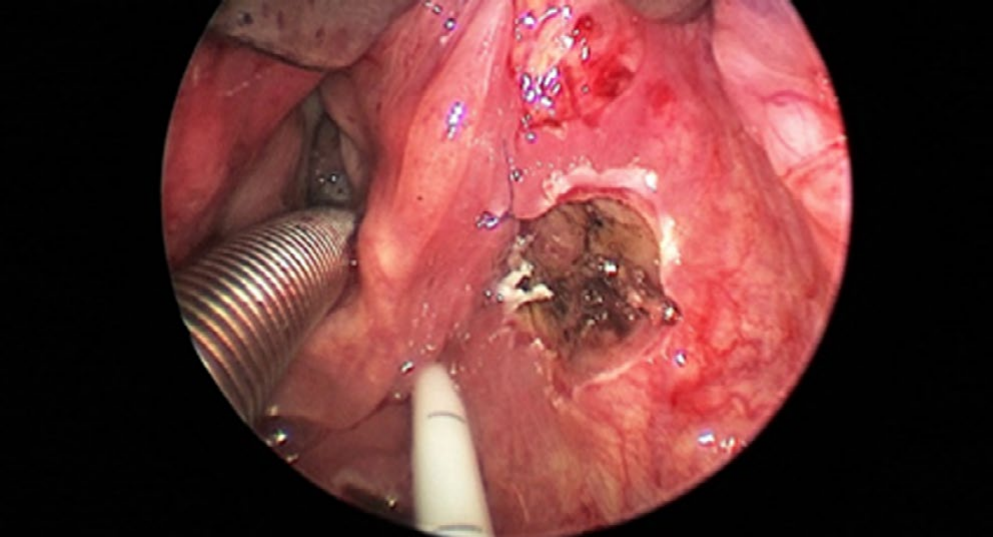
Intraoperative endoscopic view of the surgical field after the resection

Perioperative examination allowed to show that the tumor was originating from the lateral wall of the right pyriform sinus without extension to the esophagus (Figure 3). The resection was performed in piecemeal technique, to obtain a clear view of the base of implant of the tumor that was removed in macroscopic healthy margins (Figure 4). No major bleeding was observed during the procedure, and no tracheostomy was considered necessary. A nasogastric feeding tube was inserted intraoperatively under endoscopic view.

**Figure 5:**
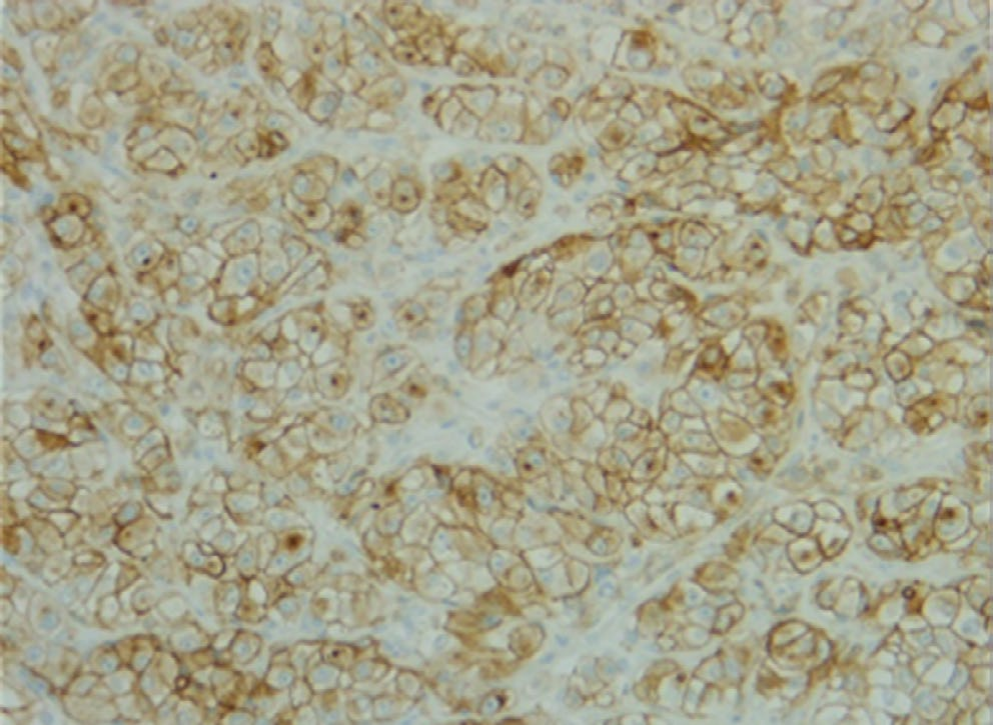
Histology of the lesion showing CD‐10 + immunohistochemical staining (original magnification 20×)

**Figure 6:**
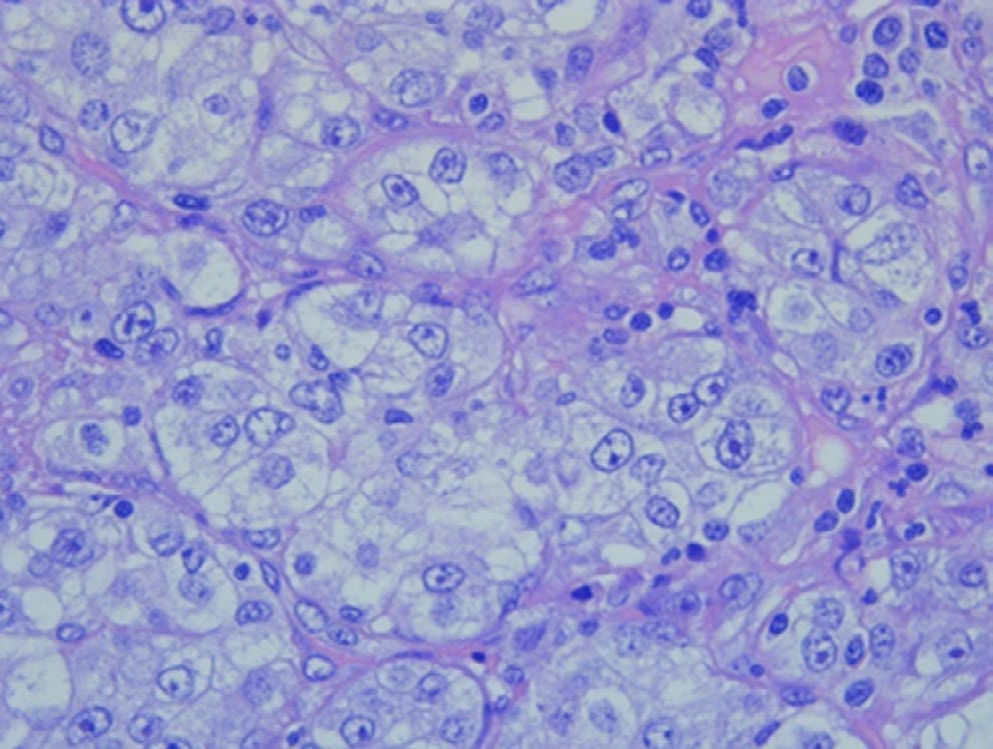
Histology of the lesion with hematoxylin and eosin stain (original magnification 40×)

Immunohistochemistry was positive for CKEA1A3, CD10, PAX8, and vimentin and negative for CK7, CK20, EMA, PAX2, and cKit, allowing the diagnosis of metastasis of pRCC (Figures 5and 6).

The postoperative outcomes were regular, and the patient was discharged 7 days after surgery under antibiotics (cefpodoxime 200 mg twice a day for 7 days and cold soft diet). After 1 month, the fibrolaryngoscopy showed an optimal healing of the mucosa, no laryngeal palsy or other anomalies of the larynx and the pharynx. The patient died 4 months later because of extra‐laryngeal complications of the renal disease.

## 3 DISCUSSION

Renal cell carcinoma is the ninth most common cancer worldwide, and it represents 2%‐3% of all malignant tumors in adults. It is not a single entity, but rather a collection of different types of tumors, each derived from the various parts of the nephron (clear cell 75%; papillary 15%; chromophobe 5%; and other <5%). RCC incidence increases markedly with age and is associated with male gender, excess body weight, hypertension, and cigarette smoking.^2^, ^3^, ^4^ Seventy percent of RCC develop metastatic disease with an average 5‐year survival rate of 5%‐15%. Approximately 30% of patients with RCC will show metastatic disease at initial diagnosis, and the most common sites of metastasis are lungs (50%‐60%), liver (30%‐40%), bones (30%‐40%), suprarenal glands, pleura, brain (5%), and soft tissues.^4^, ^5^ Head and neck are uncommon sites of metastasis (8%‐15% of all metastasis from RCC), and only 1% of patients have RCC metastasis confined exclusively to this region.^6^ Head and neck localization of RCC metastasis reported in literature are the neck nodes (48%), the paranasal sinuses (34%), the thyroid gland (14%), the skull bone (10%), the parotid gland (5%), the tongue (5%), the facial skin (5%), and other subsites (15%) including larynx, infratemporal fossa, parathyroid, and masseteric space.^7^, ^8^

The most frequent malignancy observed in the piriform sinus is the squamous cell carcinoma; other primary and metastatic hypopharyngeal tumors are exceedingly rare and include minor salivary gland tumors, mesenchymal neoplasms, and metastases from melanoma, lung and breast cancer,^9^ while, although RCC is the third most common cause of distant metastasis to the head and neck, the involvement of the hypopharynx by RCC is extremely rare. To the best of our knowledge, only one case of metastasis from RCC to the pyriform sinus has been reported so far.^10^ Metastases that do not seem to follow normal hematogenous flow patterns may be explained by a right‐to‐left heart shunt, spontaneous regression of the lung disease, or microscopic seeding of the lung parenchyma. When there is no evidence of lung or liver disease, the head and neck isolated localization can be explained by the neoplastic spread through Batson’s venous plexus or through the thoracic duct.^6^

Symptoms of secondary hypopharyngeal tumors do not differ substantially from those of other malignant lesions, except for hemoptysis, which is sometimes the heralding symptom of a metastatic renal cell carcinoma due to the high vascular stroma of RCCs, as observed in our case.^11^

Head and neck CT scan with contrast medium showed a paramount utility revealing the vascular pattern of the lesion, and as a consequence, a preoperative out‐patient biopsy was avoided.^12^

Treatment of RCC metastasis should be evaluated according to the local and systemic spread of the disease, and the prognosis *quod vitam* of the patients: patients with solitary metastatic lesions of renal cell carcinoma following nephrectomy results in a 41% survival at 2 years and 13% survival at 5 years.^13^ As a consequence, the surgical treatment is indicated mainly to improve the quality of life or to treat severe symptoms and must be as much conservative as possible, like in the present case.

In literature, pharyngo‐laryngeal RCC metastasis have been treated through nonsurgical and surgical endoscopic/open approaches.^4^, ^11^, ^14^, ^15^, ^16^, ^17^, ^18^

Renal cell carcinoma is traditionally described as a radioresistant tumor, but different studies have reported good site‐specific response to radiation therapy in case of bone and soft‐tissue metastasis,^6^ and during the past 10 years, a number of targeted therapeutic and immunotherapy agents have been approved for the treatment of metastatic RCC.^3^

Deep cervical metastasis is generally treated by open approach, while large hypopharyngeal tumors may impair airways and could require salvage procedures such as tracheostomy.

Preoperative embolization could be helpful in preventing massive hemorrhage during the resection of bigger and deeper lesions,^6^ but it was not considered necessary in our case due to the limited vascular supply of the tumor that was controlled with monopolar cautery and metallic clips.

Debulking of the lesions is a palliative option but it could be burdened by a limited and temporary improvement of the quality of life and should be considered only when radical resection is not feasible or could require invasive and challenging procedures that could be considered as an over‐treatment in case of poor prognosis.

In our case, although the patient presented poor prognosis due to the systemic spread of the disease, the pharyngeal metastasis was associated with a life‐threatening respiratory impairment. Tracheostomy was considered as a possible palliative option, but the tumor showed to be pedicled, and a conservative endoscopic approach by an orotracheal intubation was not considered contraindicated. TLM allowed the radical resection of the mass with minimal intraoperative bleeding and low thermal injury of the surrounding tissues. The patient recovered within few days a physiological breathing and swallowing and did not experience further pathologies of the larynx and the pharynx.

## 4 CONCLUSION

Renal cell carcinoma distant metastasis to the head and neck should be suspected when patients with a clinical history of RCC show a head and neck lesion; in such cases, surgery aims to offer a better quality of life despite the poor long‐term survival outcomes. TLM allows for a precise and bleeding‐less radical resection, with low morbidity and high improvement of the quality of life, avoiding expensive and invasive procedures such as chemotherapy, radiotherapy, and tracheostomy.

## CONFLICT OF INTEREST

The authors declare no conflicts of interest.

## AUTHOR CONTRIBUTIONS

EG and FC were involved in patient management and wrote the manuscript. CM provided final editing and review of the manuscript. CG provided molecular analysis of samples. RP was involved in patient management and provided editing and review of the manuscript.

## ETHICAL APPROVAL

The research did not involve any animal models; the research involved human participants in accordance with the ethical standards of the institutional and/or national research committees and with the 1964 Helsinki Declaration and its later amendments or comparable ethical standards; and informed consent was obtained from all individual participants included in the study.

## References

[1] Miyamoto R , Helmus C . Hypernephroma metastatic to the head and neck. Laryngoscope. 1973;83:898‐905. 4711327 10.1288/00005537-197306000-00007

[2] Jonasch E , Gao J , Rathmell WK . Renal cell carcinoma. BMJ. 2014;349:g4797. 25385470 10.1136/bmj.g4797 PMC4707715

[3] Hsieh JJ , et al. Renal cell carcinoma. Nat Rev Dis Primers. 2017;3:17009p. 28276433 10.1038/nrdp.2017.9 PMC5936048

[4] Mehdi IJ , et al. Renal Cell Carcinoma metastasizing to larynx: a case report. Gulf J Oncol. 2012;1:70‐74. 22227550

[5] Langille G , Taylor SM , Bullock MJ . Metastatic renal cell carcinoma to the head and neck: summary of 21 cases. J Otolaryngol‐Head and Neck Surg. 2008;37:515‐521. 19128586

[6] Pritchyk KM , Schiff BA , Newkirk KA , Krowiak E , Deeb ZE . Metastatic renal cell carcinoma to the head and neck. Laryngoscope. 2002;112:1598‐1602. 12352670 10.1097/00005537-200209000-00012

[7] Liou T‐N , Scott‐Wittenborn NR , Kallogjeri D , Lieu JE , Pipkorn P . Survival of renal cell carcinoma metastatic to nonthyroid head and neck region: a systematic review. Laryngoscope. 2018;128:889‐895. 28988421 10.1002/lary.26892

[8] Lieder A , Guenzel T , Lebentrau S , Schneider C , Franzen A . Diagnostic relevance of metastatic renal cell carcinoma in the head and neck: an evaluation of 22 cases in 671 patients. Int Braz J Urol. 2017;43:202‐208. 27649110 10.1590/S1677-5538.IBJU.2015.0665 PMC5433357

[9] Barnes L . Metastases to the head and neck: an overview. Head Neck Pathol. 2009;3:217‐224. 20596975 10.1007/s12105-009-0123-4 PMC2811631

[10] Chauhan S , Yadav SS , Tomar V . Renal cell carcinoma presenting as Dysphagia. Indian J Surg. 2015;77:153‐155. 25972679 10.1007/s12262-015-1216-9 PMC4425783

[11] Nicolai P , Puxeddu R , Cappiello J , et al. Metastatic neoplasms to the larynx: report of three cases. Laryngoscope. 1996;106:851‐855. 8667982 10.1097/00005537-199607000-00013

[12] Ishak AI , et al. Multiple metastatic deposits in the head and neck region from a renal cell carcinoma. Malays J Med Sci. 2010;17:71‐74. 22135565 PMC3216188

[13] Gottlieb MD , Roland JT . Paradoxical spread of renal cell carcinoma to the head and neck. Laryngoscope. 1998;108:1301‐1305. 9738745 10.1097/00005537-199809000-00007

[14] Greenberg RE , Cooper J , Krigel RL , Melvyn Richter R , Kessler H , Petersen RO . Hoarseness; a unique clinical presentation for renal cell carcinoma. Urology. 1992;40:159‐161. 1502755 10.1016/0090-4295(92)90518-2

[15] Dee SL , Eshghi M , Otto CS . Laryngeal metastasis 7 years after radical nephrectomy. Arch Pathol Lab Med. 2000;124:1833‐1834. 11100069 10.5858/2000-124-1833-LMYARN

[16] Sarkis P , Bou‐Malhab F , Mouaccadieh L . Solitary laryngeal metastasis from renal cell carcinoma of the kidney: clinical case and review of the literature. Progrés en Urologie. 2012;22:307‐309. 22515928 10.1016/j.purol.2011.11.002

[17] Demir L , Erten C , Somali I , et al. Metastases of renal cell carcinoma to the larynx and thyroid: two case reports on metastasis developing years after nephrectomy. Can Urol Assoc Journal. 2012;6:209‐212. 10.5489/cuaj.11255 PMC3478395 23093648

[18] Campos Alvarez C , et al. Endobronchial and laryngeal metastasis. An unusual form of dissemination of the hypernephroma. Arch Esp Urol. 2000;53:734‐736. 11126979

